# Differential regulation of myofibrillar proteins in skeletal muscles of septic mice

**DOI:** 10.14814/phy2.14248

**Published:** 2019-10-29

**Authors:** Vanessa Moarbes, Dominique Mayaki, Laurent Huck, Philippe Leblanc, Theodoros Vassilakopoulos, Basil J. Petrof, Sabah N. A. Hussain

**Affiliations:** ^1^ Meakins‐Christie Laboratories and Translational Research in Respiratory Diseases Program Research Institute of the McGill University Health Centre Montréal Québec Canada; ^2^ Department of Critical Care McGill University Health Centre Montréal Québec Canada; ^3^ Critical Care Department, National & Kapodistrian University of Athens, Medical School, Evgenideion Hospital Athens Greece; ^4^ Department of Medicine McGill University Health Centre Montréal Québec Canada

**Keywords:** sepsis, muscle atrophy, proteasome, autophagy, myofibrillar proteins, myosin, actin, Troponin, tropomyosin

## Abstract

Sepsis elicits skeletal muscle atrophy as a result of decreased total protein synthesis and/or increased total protein degradation. It is unknown how and whether sepsis differentially affects the expression of specific myofibrillar proteins in respiratory and limb muscles. In this study, we measured the effects of sepsis myofibrillar mRNAs and their corresponding protein levels in the diaphragm (DIA) and tibialis anterior (TA) muscles in a murine cecal ligation and perforation (CLP) model of sepsis. Male mice (C57/BL6j) underwent CLP‐induced sepsis. Sham‐operated mice were subjected to the same surgical procedures, except for CLP. Mice were euthanized 24, 48, or 96 h postsurgery. Transcript and protein levels of autophagy‐related genes, ubiquitin E3 ligases, and several myofibrillar genes were quantified. Sepsis elicited transient fiber atrophy in the DIA and prolonged atrophy in the TA. Atrophy was coincident with increased autophagy and ubiquitin E3 ligase expression. Myosin heavy chain isoforms decreased at 24 h in the DIA and across the time‐course in the TA, myosin light chain isoforms decreased across the time‐course in both muscles, and troponins T and C as well as tropomyosin decreased after 24 and 48 h in both the DIA and TA. α‐Actin and troponin I were unaffected by sepsis. Sepsis‐induced decreases in myofibrillar protein levels coincided with decreased mRNA expressions of these proteins, suggesting that transcriptional inhibition is involved. We hypothesize that sepsis‐induced muscle atrophy is mediated by decreased transcription and enhanced degradation of specific myofibrillar proteins, including myosin heavy and light chains, troponin C, troponin T, and tropomyosin.

## Introduction

An important manifestation of severe sepsis in critically ill patients is the development of skeletal muscle weakness and atrophy (Callahan and Supinski, 2009). In the ventilatory muscles, short‐term ramifications of sepsis‐induced weakness include difficult weaning from mechanical ventilation and increased risk of recurrence of respiratory failure after recovery from sepsis (Dres et al., 2017). In the limb muscles, sepsis can lead to prolonged weakness that leads to functional impairment, limited physical activity, and poor quality of life (Cheung et al., 2006; Dos et al., 2016).

Skeletal muscle mass is maintained by a balance between anabolic protein synthesis and catabolic protein degradation. Protein degradation is accomplished via four metabolic systems: the calpain, caspase‐3, ubiquitin‐proteasome (UPS), and autophagy‐lysosome (ALP) pathways. The calpain, caspase, and UPS pathways are responsible for degrading myofibrillar proteins into smaller peptides. The ALP is responsible for the degradation of cytoplasmic proteins and organelles, including the mitochondria and peroxisomes. Because of significant increases in calpain and caspase‐3 pathway activities, muscle protein ubiquitination, proteasome activity, and autophagy‐lysosome pathway activation have all been observed in the ventilatory and limb muscles of septic humans and animals, sepsis‐induced skeletal muscle atrophy has been mainly attributed to increased protein degradation (Hobler et al., 1999; Klaude et al., 2007; Supinski et al., 2009; Mofarrahi et al., 2012). It is not clear, however, how sepsis affects the synthesis and levels of specific myofibrillar proteins. In addition to their critical importance in facilitating muscle contraction, myofibrillar proteins represent a significant portion of overall muscle fiber mass. Therefore, the first objective of this study was to measure the effects of sepsis on selective myofibrillar protein levels in the ventilatory and limb muscles over an extended time course of sepsis using a murine model of cecal ligation and perforation (CLP)‐induced sepsis. The rationale for comparing the diaphragm to a limb muscle was to determine whether myofibrillar proteins in the ventilatory muscles are differentially less sensitive to the adverse effects of sepsis as compared to limb muscles due to their more sustained activity level. We hypothesize, based on previous studies in septic humans and experimental animals, that myofibrillar protein levels in the ventilatory muscles are relatively spared from the inhibitory effects of sepsis in comparison to the limb muscles (Tiao et al., 1997; Morel et al., 2017; Stana et al., 2017; Talarmin et al., 2017).

Along with increased protein degradation, sepsis‐induced skeletal muscle atrophy is also associated with inhibited protein synthesis. Protein synthesis is regulated by transcriptional, epigenetic, and post‐transcriptional mechanisms, and early reports suggest that its inhibition in septic limb muscles is regulated by post‐transcriptional events that involve protein translation (Vary and Kimball, 1992; Lang et al., 2000). There is also evidence that the levels and activities of positive regulators of protein synthesis, such as insulin‐like growth factor 1 (IGF‐1), protein kinase B (AKT), and mammalian target of rapamycin complex 1 (mTORC1), diminish in skeletal muscles in response to sepsis (Lang et al., 2000; Lang et al., 2007; Stana et al., 2017). However, the precise roles that sepsis plays in the synthesis of myofibrillar proteins in these muscles remain unclear. A second objective of our study, therefore, was to explore the role of transcriptional downregulation of α‐Actin, myosin, troponin, and tropomyosin isoforms in ventilatory and limb muscle atrophy by measuring their mRNA transcript levels over an extended time course of sepsis. We hypothesize that sepsis elicits significant decreases in the synthesis of myofibrillar genes and that these decreases contribute to the reduction in myofibrillar protein levels in the ventilatory and limb muscles.

## Methods

### Materials

Antibodies for α‐Actin, troponin T, α‐tropomyosin, β‐tropomyosin, and laminin proteins were purchased from Sigma‐Aldrich Inc. Antibodies for troponin I and troponin C were purchased from Thermo Scientific Inc. Antibodies for β‐TUBULIN; myosin heavy (MHC) and light chain (MLC) isoforms were obtained from the University of Iowa Developmental Studies Hybridoma Bank.

### Cecal ligation and perforation

All procedures were approved by the Animal Ethics Committees of McGill University and were in accordance with the guidelines of the Canadian Council of Animal Care. Adult (7‐ to 8‐wk‐old) male wild‐type C57/Bl6j mice were briefly anesthetized using an isoflurane (3.5 to 4.5%) vaporizer. A midline laparotomy was performed on each animal. The cecum was carefully isolated, ligated with a size 4‐0 silk tie approximately 1 cm from the cecal tip, and punctured with a 26½ gauge needle. Punctures were made in one pass, through both sides of the bowel wall, allowing the extrusion of a small amount of fecal material into the peritoneal cavity. The abdominal cavity was closed in two separate layers with size 3–0 absorbable polyfilament sutures in an interrupted pattern. Sham‐operated mice were subjected to the same procedures, except for cecum ligation and puncture. All animals received subcutaneous injections of buprenorphine (0.05 to 0.2 mg/kg in 0.9% saline) immediately after surgery and every 24 h thereafter to minimize pain. Animals were returned to their cages (3–5 mice per cage) post‐surgery. They had free access to food and water and were closely monitored twice per day for signs of excessive pain or distress, including lack of movement, agonized breathing, or excessive body weight loss (20%). Any mouse determined to be moribund was euthanized. Mice were euthanized 24, 48, or 96 h post‐surgery and the diaphragm (DIA) and tibialis anterior (TA) muscle from each animal was quickly excised and prepared for cross‐sectional area measurements or frozen in liquid nitrogen and stored at <−80°C for use in mRNA extraction and immunoblotting.

### Indirect calorimetry

Indirect calorimetry was measured at the McGill Mouse Metabolic Platform. Animals were individually housed in PhenoMaster metabolic cages (TSE Systems) and acclimatized for three days before the commencement of experimentation. Baseline O_2_ consumption, CO_2_ production, energy expenditure (heat), respiratory exchange ratio (RER), and locomotion activity (beam breaks) were measured two days prior to and four consecutive days after sham or CLP surgery. All average daily metabolic parameters were normalized to daily body weight of mice. Locomotion activity was simultaneously monitored using infrared sensor frames. The RER was calculated as CO_2_ production divided by O_2_ consumption. Energy expenditure was calculated as (VO_2_*[3.815 + (1.232*RQ)]*4.1868).

### Muscle fiber atrophy

TA muscles were mounted on plastic blocks and frozen in liquid nitrogen‐cooled isopentane. Costal parts of diaphragms were removed intact, positioned flat in OCT compound on a piece of plastic, and then frozen in liquid nitrogen‐cooled isopentane. Frozen muscles were positioned vertically on a cryostat to obtain cross‐sections of each sample, cut into 10 μm‐thick sections, fixed in paraformaldehyde (2%) in phosphate buffered saline (PBS) for 20 min, then permeabilized in PBS/Triton (0.2%) for 10 min. Sections were washed with PBS/glycine (100mM) then blocked with PBS/bovine serum albumin (2%)/Triton (0.2%)/Tween (0.05%) for 30 min. They were then incubated with a primary laminin antibody (L9393, at 1:750) at room temperature for 1 h to outline muscle fibers, then washed three times with PBS/Triton (0.2%)/Tween (0.05%), and incubated with Alexa Fluor®568 secondary antibody at room temperature for 1 h. Following three more washes with the PBS/Triton/Tween solution, sections were counterstained with 4’,6‐diamino‐2‐phenylindole (0.5 ng/mL) in PBS at room temperature for 5 min, rinsed twice with double distilled water, then slide‐mounted with Lerner AquaMount™ mounting medium. Images were captured using an Olympus IX70 microscope (20X magnification). Cross‐sectional areas of at least 600 fibers per muscle cross‐section were measured in non‐overlapping fields with ImageJ software (US National Institutes of Health). Measurements were performed by a single observer who was blinded to the identity of the samples.

### Transmission electron microscopy (TEM)

Animals that were euthanized 48 h post‐sham or ‐CLP surgery were first perfused through the left ventricle with normal saline then with a sodium cacodylate‐buffered (01.M, pH 7.4) glutaraldehyde (3%) fixative. DIA and TA muscles were excised, dissected into segments, and placed in fresh fixative at room temperature for 2 h. Fixed tissues were washed in cacodylate buffer overnight at 4ºC, then sequentially treated with OsO4 (1%) in buffer, tannic acid (2%) in buffer, and uranyl acetate (2%) in distilled water. Tissues were dehydrated using a graded series (methanol to propylene oxide) of solvents then infiltrated with and embedded in epoxy resin. Ultrathin sections (60 nm) were counterstained with methanolic uranyl acetate and lead citrate and imaged using a FEI Tecnai™ 12 transmission electron microscope at 120 kV. Digitally‐captured images were analyzed for the presence of autophagosomes, defined as vesicle compartments with double limiting membranes containing heterogeneous cytosolic materials. Analysis was performed by a single observer who was blinded to the identity of the samples.

### RNA extraction and ‐time PCR

Total RNA was extracted from muscle samples using a GenElute™ Mammalian Total RNA Miniprep Kit (Sigma‐Aldrich). Quantification and purity of RNA was assessed using the A260/A280 absorption method. Total RNA (2 μg) was reverse transcribed using a Superscript II® Reverse Transcriptase Kit and random primers (Invitrogen Canada). Reactions were incubated at 42°C for 50 min and at 90°C for 5 min. Real‐time PCR detection of mRNA expression was performed using a Prism® 7000 Sequence Detection System (Applied Biosystems). Specific primers (10 μmol/L) were designed to quantify: sarcomeric Actin (α‐Actin); isoforms of myosin heavy chain (MHC), myosin light chain (MLC), troponin T, troponin I, troponin C, and tropomyosin (Table 1). Specific primers were also designed to quantify three autophagy‐related genes (Lc3b, Sqstm1, and Bnip3), the transcription factor Foxo1, and three proteasome‐related ubiquitin E3 ligases (Fbxo32, Trim63, and Trim32) that are essential to the ubiquitination of skeletal myofilament proteins. Fbxo32 and Trim63 are also known as atrogin‐1 and MuRF1 respectively. Cyclophilin B served as an endogenous control transcript. Each primer (3.5 μL) was combined with reverse‐transcriptase reagent (1 μL) and SYBR® Green Master Mix (25 μL) (Qiagen). The thermal profile was as follows: 95°C for 10 min; 40 cycles each of 95°C for 15 sec; 57°C for 30 sec; and 72°C for 33 sec. All real‐time PCR experiments were performed in triplicate. A melt analysis for each experiment was performed to assess primer‐dimer formation or contamination. Cycle threshold (CT) values were obtained for each target gene. ΔCT values (normalized gene expression) were calculated as C_T_ of target gene minus C_T_ of cyclophilin B. Relative mRNA level quantifications of target genes were determined using the threshold cycle (ΔΔ^CT^) method. Absolute mRNA levels were calculated as 2^−ΔCT^.

**Table 1:** Primers used for real‐time PCR experiments to detect mRNA expression of various myofilament genes and E3 ligases in the DIA and TA of Sham and CLP groups.

Name	Primers
Bnip3	F‐TTCCACTAGCACCTTCTGATGA R‐GAACACCGCATTTACAGAACAA
Cyclophilin B	F‐ATGGCACAGGAGGAAAGAGC R‐ATGATCACATCCTTCAGGGG
Fbxo32 (Atrogin‐1)	F‐TGGGTGTATCGGATGGAGAC R‐TCAGCCTCTGCATGATGTTC
Foxo1	F‐ AAGGATAAGGGCGACAGCAA R‐ TGGATTGAGCATCCACCAAG
Map1(Lc3b)	F‐CGATACAAGGGGGAGAAGCA R‐ACTTCGGAGATGGGAGTGGA
MHCIIx	F‐GCGAATCGAGGCTCAGAACAA R‐GTAGTTCCGCCTTCGGTCTTG
MHCIIa	F‐ACTTTGGCACTACGGGGAAAC R‐CAGCAGCATTTCGATCAGCTC
MHCIIb	F‐TTTGCTTACGTCAGTCAAGGT R‐AGCGCCTGTGAGCTTGTAAA
MHCI	F‐ CCTGCGGAAGTCTGAGAAGG R‐ CTCGGGACACGATCTTGGC
MLC1(1f)	F‐AACACTCTGGGTCCACCCTC R‐TGACACTTGGAAGAGCAGTGTGA
MLC1(3f)	F‐TGC TGA CCA GAT TGC CGA CT R‐AGG ACG TCT CCC ACC TGA CT
MLC1(s)	F‐TTCGGGAAGGAGTGGTTCGG R‐GCAGGGGCCAGGAAAGACTA
Tpm1	F‐GGCTGAGCTCTCAGAAGGCA R‐TCAGCCCGAGTTTCAGCCTC
Tpm2	F‐GAGCACCAGCTAGCCACGTT R‐GGGCTTCCGGAGTAGAAGAGC
Tpm3	F‐TGGACCACGCCCTCAATGAC R‐GAATCCAGAGCGAGTGGGGT
Trim6*3* (MuRF1)	F‐AGAAGCTGGGCTTCATCGAG R‐TGCTTGGCACTTGAGAGGAA
Trim32	F‐CAGAGTGAGGTGCTGGTTGC R‐GGCTCCAAGGAAGCTTAGCA
Troponin T(f)	F‐AACAGATTGGCGGAGGAGAA R‐TTGGCCAGGTAGCTGCTGTA
Troponin T(s)	F‐AGCGCTTCAGAACGGAAAAG R‐GGCGTCATCCTCTGCTCTCT
Troponin I(f)	F‐TGCAGAAGAGCAGCAAGGAG R‐GGTCCCGTTCCTTCTCAGTG
Troponin I(s)	F‐GGAGTGTTGGGAGCAGGAAC R‐GAGCTCTCGGCACAAGTCCT
Troponin C(f)	F‐CCTTTGACATGTTCGATGCTG R‐TCGATGATGGCATCCAATTC
Troponin C(s)	F‐CCTGAGGAGCTGCAGGAGAT R‐TTCCCTTTGCTGTC TCCTT
α‐Actin	F‐CACTTCCTACCCTCGGCACC R‐GTAGGAGAGCACCGGCTTGT
Sqstm1(p62)	F‐GCACCTGTCTGAGGGCTTCT R‐GCTCCAGTTTCCTGGTGGAC

### Immunoblotting

Frozen DIA and TA tissue samples were homogenized in homogenization buffer (tris‐maleate (10 mmol/L), EDTA (3 mmol/L), sucrose (275 mmol/L), DTT (0.1 mmol/L), leupeptin (2 μg/mL), PMSF (100 μg/mL), aprotinin (2 μg/mL), and pepstatin A (1 mg/100 mL, pH 7.2)), then centrifuged at 500 *g* for 10 min in a cold room. Pellets were discarded, and supernatants were designated as crude homogenate. Total muscle protein in each sample was determined using the Bradford protein assay technique. Crude homogenate (25–50 μg/sample) was mixed with SDS sample buffer, boiled at 95°C for 8 min, loaded onto tris‐glycine sodium dodecyl sulfate polyacrylamide gels and electrophoretically separated into proteins that were then electrophoretically transferred to polyvinylidene difluoride (PVDF) membranes and blocked with bovine serum albumin (1%) or milk at room temperature for 1 h. PVDF membranes were incubated overnight with primary antibodies (Table 2) at 4°C, washed, then incubated with horseradish peroxidase‐conjugated secondary antibody. Specific proteins were detected using an enhanced chemiluminescence kit. Equal loading of proteins was confirmed by stripping each membrane and re‐probing with anti‐β‐TUBULIN antibody. Immunoblots were scanned with an imaging densitometer. Optical densities (OD) of protein bands were quantified using Gel‐Pro Analyzer software (MediaCybernetics).

**Table 2:** Characteristics of primary antibodies used to detect various myofibrillar proteins.

Antibody	Detected protein	Supplier
MF20	MHCI, IIA, IIB, IIX	DSHB
10F5	MHCIIB	DSHB
2F7	MHCIIA	DSHB
F310	MLC1(f)	DSHB
T14	MLC1 (s,f), MLC2(s,f)	DSHB
S21	MLC1(s)	DSHB
α‐Actin	α‐Actin	Sigma‐Aldrich
Troponin T	Troponin T(s,f)	Sigma‐Aldrich
Troponin C	Troponin C(s,f)	ThermoFisher
Troponin I	Troponin I(s,f)	ThermoFisher
Tropomyosin	Tropomyosin (α,β)	Sigma‐Aldrich
β‐TUBULIN	β‐TUBULIN	DSHB

DSHB, Developmental Studies Hybridoma Bank (U. of Iowa); MHC, myosin heavy chain; MLC, myosin light chain; f, fast; s, slow.

### Statistical analyses

Statistical analyses were performed using a SAS statistical package (SAS Institute) and SigmaStat software. Normal distribution was tested with the D’Agostino‐Pearson omnibus normality test. All data are expressed as means ± standard error of the mean. Comparisons of sham and CLP groups at a given time point were performed using a One‐Way Analysis of Variance (ANOVA) test, followed by a Tukey post‐hoc test. For non‐Gaussian distributions, a non‐parametric Kruskal–Wallis test by ranks was performed, followed by a Dunn’s multiple comparison tests. The α level was set at 0.05 for all tests and was accordingly adjusted using the sharper Bonferroni‐type procedure for multiple comparisons as described by Hochberg and Benjamini (Hochberg and Benjamini, 1990).

## Results

### Indirect calorimetry

**Figure 1:**
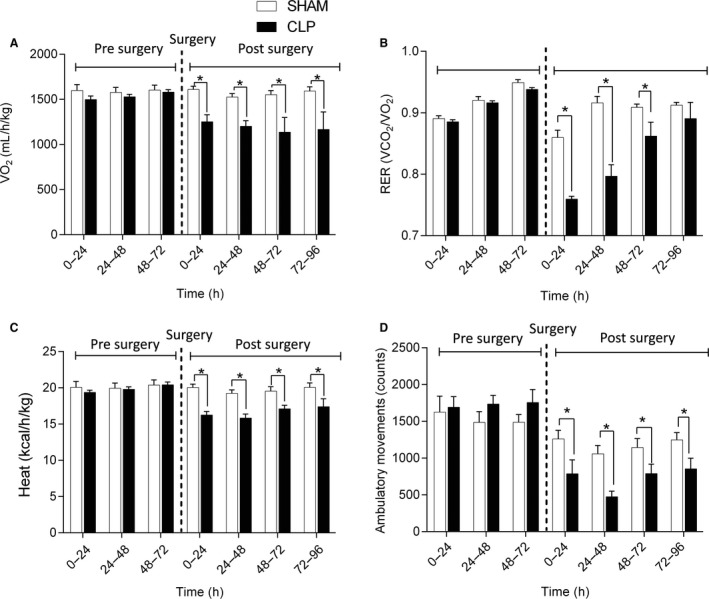
Rates of O_2_ consumption (A), respiratory exchange ratios (B), rates of heat production (C), and ambulatory movement (D) of mice in the sham and CLP groups. Values were measured for 4 days prior to and 4 days after surgical procedure. *N* = 8 per group. *P < 0.05, compared to sham group.

Over the time course of sepsis, O_2_ consumption rates, heat production rates, and ambulatory movements decreased by the 24, 48, 72, and 96 h points (Fig. 1). Respiratory exchange ratios decreased by the 24, 48, and 72 h points, but had returned to normal by 96 h (Fig. 1).

### Muscle fiber atrophy

**Figure 2:**
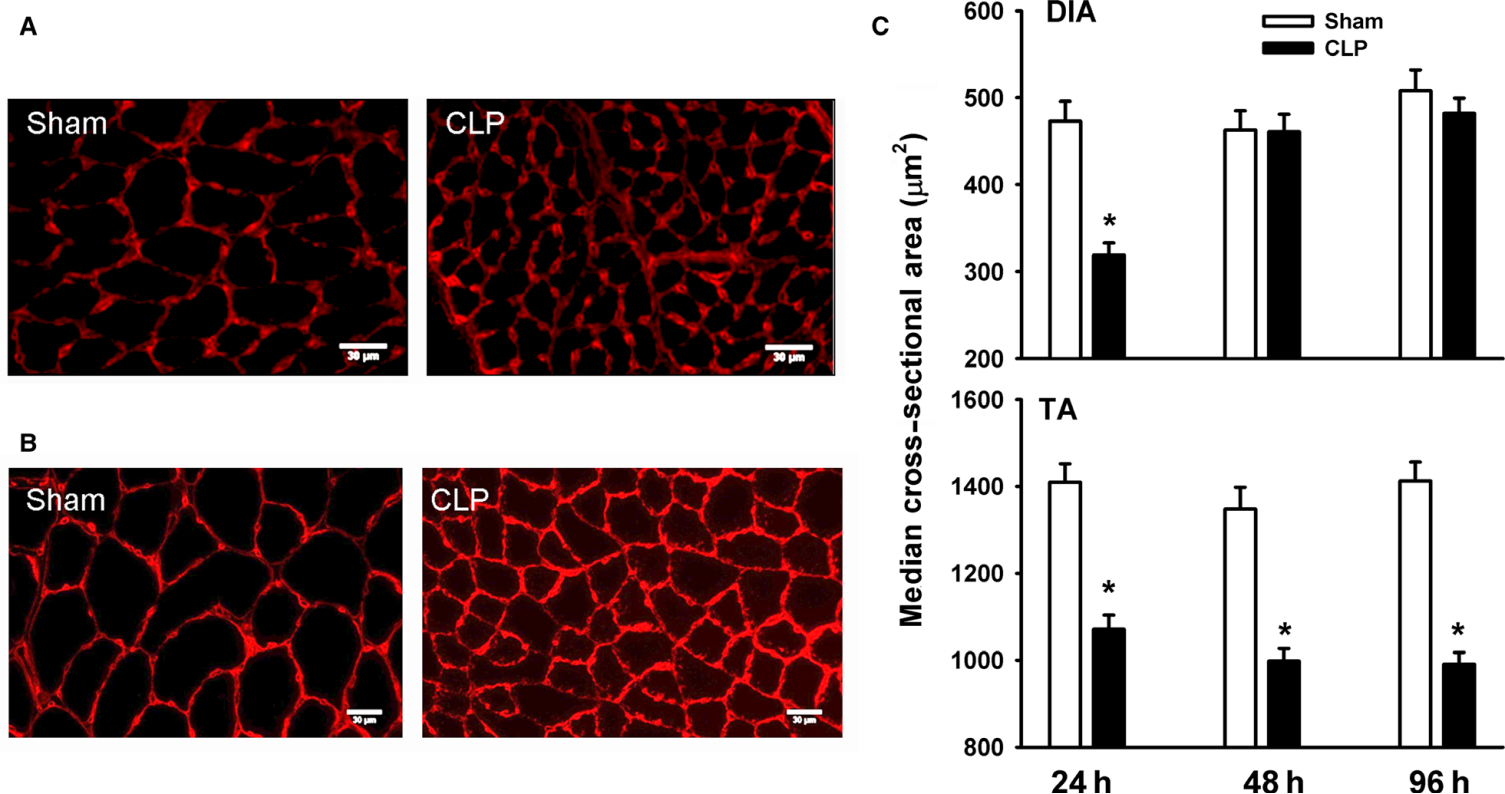
(A and B) Representative immunostaining of laminin protein in DIA (A) and TA (B) muscles of sham and CLP groups. (C) Median value of cross‐sectional areas of DIA and TA muscle fibers of sham and CLP groups. Values are means ± SEM. *P < 0.05, compared to sham. *N* = 8 per group.

DIA muscle fiber size in the CLP group transiently decreased after 24 h of sepsis (Fig. 2). There were no differences between the sham and CLP groups in DIA fiber size after 48 and 96 h of sepsis. In contrast, TA fiber size in the CLP group consistently decreased after 24, 48, and 96 h of sepsis (Fig. 2).

### Regulation of autophagy and proteasome pathways

**Figure 3:**
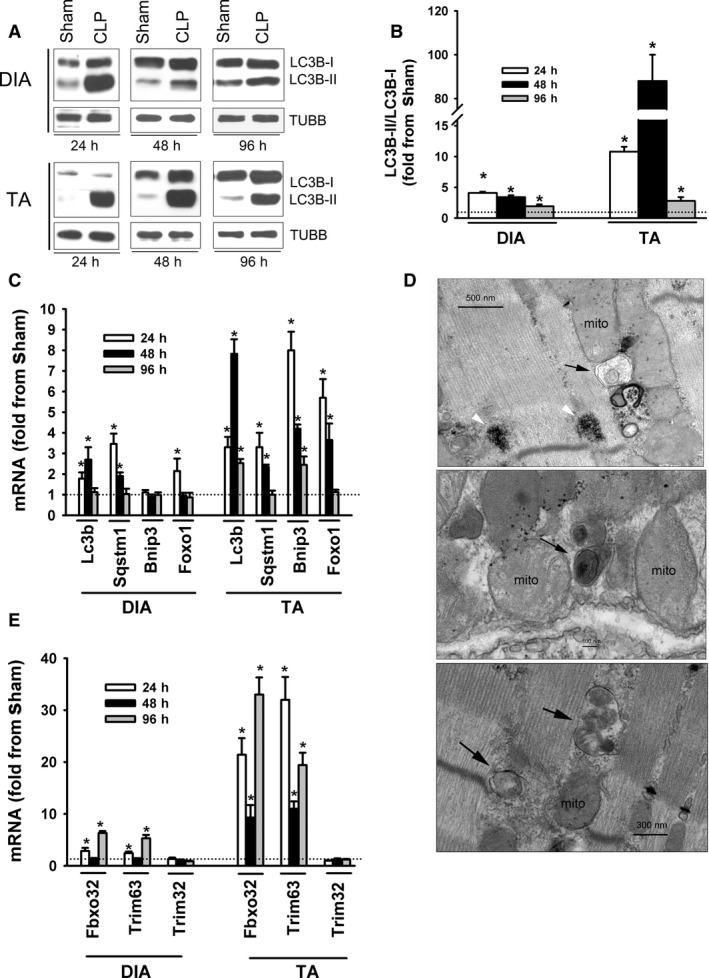
(A and B) Representative immunoblots of LC3B‐I, LC3B‐II, and β‐TUBULIN (TUBB) proteins and optical densities (OD) of LC3B‐II/LC3B‐I ratios in DIA and TA muscles of sham and CLP groups. Values are means ± SEM. *P < 0.05, compared to sham. *N* = 6 per group. (C) mRNA expressions of autophagy‐related genes in DIA and TA muscles of sham and CLP groups. Values are means ± SEM. *P < 0.05, compared to sham. *N* = 6 per group. (D) Representative electron micrographs of autophagosomes (black arrows) in DIA (bottom) and TA (top and middle) muscles of CLP group after 48 h of sepsis. White arrow heads in top panel indicate lipofuscin inclusions, mito = mitochondria. (E) mRNA expressions of ubiquitin E3 ligases Fbxo32 (atrogin‐1), Trim63 (MuRF1), and Trim32 in DIA and TA muscles of sham and CLP groups. Values are means ± SEM. *P < 0.05, compared to sham. *N* = 8 per group.

LC3B protein lipidation (LC3B‐II) intensity was used as an indirect index of autophagosome formation in skeletal muscles, as in our previous study (Stana et al., 2017). The ratio of LC3B‐II to LC3B‐I (cytosolic protein) in the DIA increased after 24 h of sepsis and remained higher relative to the sham group for as long as 48 and 96 h post‐CLP (Fig. 3A–B). LC3B‐II/LC3B‐I ratios in the TA increased after 24, 48, and 96 h of sepsis, with peak increases occurring after 48 h (Fig. 3A–C). Sepsis‐induced increases in LC3B‐II/LC3B‐I ratios were relatively higher in the TA than they were in the DIA (Fig. 3A–B). Transcript levels of three autophagy‐related genes (Lc3b, Sqstm1, and Bnip3*)* and a transcription factor that regulates their expression (Foxo1) were also measured as additional indices of autophagy. Lc3b and Sqstm1 increased in the DIA after 24 and 48 h of sepsis while Foxo1 only increased after 24 h (Fig. 3C). Sepsis had no effect on Bnip3 in the DIA. Lc3b and Bnip3 increased in the TA after 24, 48, and 96 h of sepsis, while Sqstm1 and Foxo1 increased after 24 and 48 h (Fig. 3C). Sepsis‐induced increases in Lc3b, Sqstm1, Bnip3, and Foxo1 mRNA levels were relatively higher in the TA than in the DIA (Fig. 3C). TEM imaging revealed the presence of autophagosomes containing diverse cargo in both the DIA and TA of the CLP group (Fig. 3D).

Transcript levels of three E3 ubiquitin ligases (Fbxo32, Trim63, and Trim32) were measured as an index of activation of the UPS. Fbxo32 (atrogin‐1) and Trim63 (MuRF1) mRNA levels increased in the DIA after 24 and 96 h of sepsis (Fig. 3E). Fbxo32 and Trim63 levels increased in the TA after 24, 48, and 96 h of sepsis (Fig. 3E). Trim32 levels in the DIA and TA were not affected by sepsis (Fig. 3E).

### Myosin heavy chain expression

**Figure 4:**
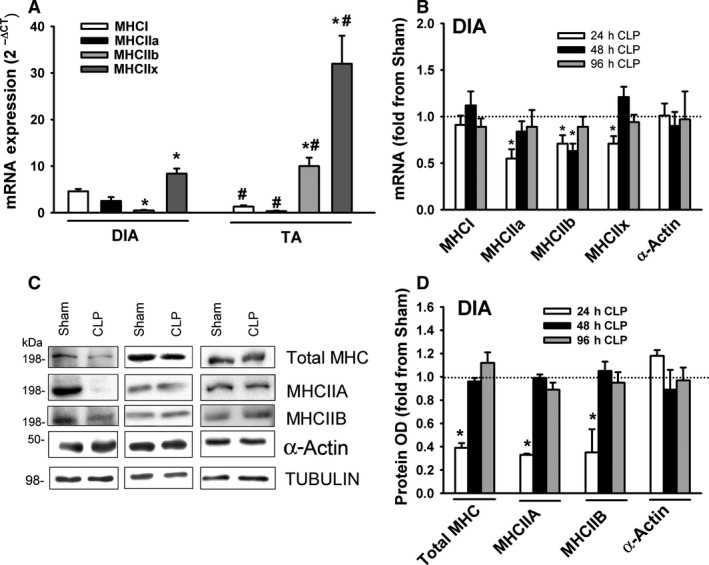
(A) mRNA levels of MHC isoforms in DIA and TA muscles of sham group. Data expressed as absolute values. *P < 0.05, compared to MHCI; #*P* < 0.05, compared to corresponding isoform in the DIA. (B) mRNA expressions of myosin heavy chain isoforms MHCI, MHCIIa, MHCIIb, MHCIIx, and α‐Actin in DIA of CLP group. Values are means ± SEM, expressed as fold change from corresponding sham group. *P < 0.05, compared to sham. *N* = 6 per group. (C) Representative immunoblots of total MHC, MCIIA, MHCIIB, α‐Actin, and β‐TUBULIN (equal loading indicator) in DIA of sham and the CLP groups. (D) Protein optical densities of total MHC, MHCIIA, MHCIIB, and α‐Actin in DIA of CLP group. Values are means ± SEM, expressed as fold change from corresponding sham group. *P < 0.05, compared to sham. *N* = 6 per group.

Mammalian skeletal muscles contain four major myosin heavy‐chain (MHC) isoforms: slow (MHCI) and fast (MHCIIa, MHCIIx, and MHCIIb). mRNA expressions of MHCI, MHCIIa, MHCIIx, and MHCIIb were detected in the DIA of the sham group (Fig. 4A). MHCIIb mRNA levels were relatively low. Sepsis had no effect on MHCI levels (Fig. 4B). MHCIIa and MHCIIx mRNA levels decreased after 24 h of sepsis, MHCIIb levels decreased after 24 and 48 h (Fig. 4B). Immunoblotting using antibodies that detect all MHC isoforms (including the low abundant MHCI) and MHCIIA and MHCIIB revealed that total MHC, MHCIIA, and MHCIIB protein levels decreased after 24 h of sepsis (Fig. 4C–D).

**Figure 5:**
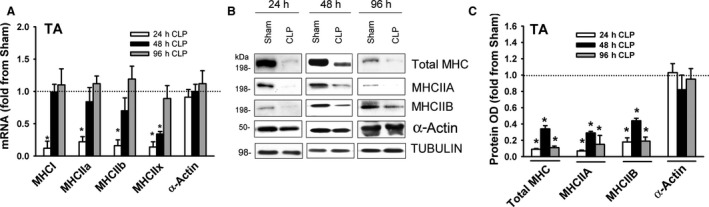
(A) mRNA expressions of myosin heavy chain isoforms MHCI, MHCIIa, MHCIIb, MHCIIx, and α‐Actin in TA of CLP group. Values are means ± SEM, expressed as fold change from corresponding sham group. *P < 0.05, compared to sham. *N* = 6 per group. (B) Representative immunoblots of total MHC, MCIIA, MHCIIB, α‐Actin, and β‐TUBULIN (equal loading indicator) in TA of sham and the CLP groups. (C) Protein optical densities of total MHC, MHCIIA, MHCIIB, and α‐Actin in TA of CLP group. Values are means ± SEM, expressed as fold change from corresponding sham group. *P < 0.05, compared to sham. *N* = 6 per group.

mRNA expressions of MHCI, MHCIIa, MHCIIx, and MHCIIb were detected in the TA of the sham group (Fig. 4A). MHCI and MHCIIa levels were relatively low. Relative levels of MHCIIb and MHCIIx mRNA in the TA were higher than in the DIA; relative levels of MHCI and MYHCIIa were lower (Fig. 4A). MHCI, MHCIIa, MHCIIb and MHCIIx levels decreased in the TA after 24 h of sepsis (Fig. 5A). Total MHC, MHCIIA, and MHCIIB protein levels decreased after 24, 48, and 96 h of sepsis (Fig. 5C–D). mRNA and protein levels of α‐Actin were not affected by sepsis in either muscle (Fig. 5A–C).

### Myosin light chain expression

**Figure 6:**
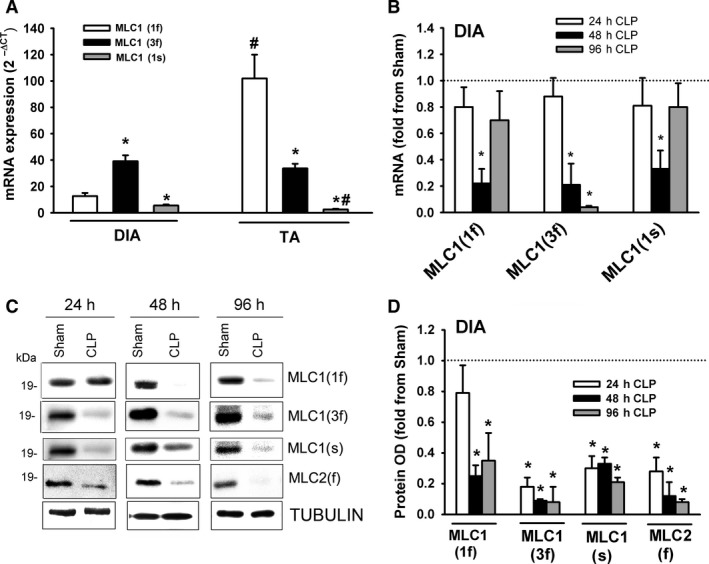
(A) mRNA levels of myosin light chain isoforms in DIA and TA muscles of sham group. Data expressed as absolute values. *P < 0.05, compared to MLC1(1f); #*P* < 0.05, compared to corresponding isoform in the DIA. (B) mRNA expressions of myosin light chain isoforms MLC1(1f), MLC1(3f), and MLC1(s) in DIA of CLP group. Values are means ± SEM, expressed as fold change from corresponding sham group. *P < 0.05, compared to sham. *N* = 6 per group. (C) Representative immunoblots of MLC1(1f), MLC1(3f), MLC1(s), MLC2(f), and β‐TUBULIN (equal loading indicator) proteins in DIA of sham and CLP groups. (D) Protein optical densities of total MLC1(1f), MLC1(3f), MLC1(s), and MLC2(f) in DIA of CLP group. Values are means ± SEM, expressed as fold change from corresponding sham group. *P < 0.05, compared to sham. *N* = 6 per group.

Two small (17–23 kDa) polypeptide units known as myosin light chains (MLCs) are associated with an MHC head‐tail junction. MLC1 is known as “essential” or “alkali” MLC and MLC2 is known as “regulatory” MLC (Schiaffino and Reggiani, 2011). These light chain units regulate the speed of contraction by playing key roles in the modulation of ATPase activity and actinomyosin interaction. MLC1 and MLC2 are differentially expressed in various fiber types and also exist in fast (MLC1(1f), MCL1(3f), MLC2(f)) and slow (MLC1(s), MLC2(s)) isoforms (Schiaffino and Reggiani, 2011). In the DIA of the sham group, mRNA expression of MLC1(3f) was relatively more abundant than MLC1(1f) or MLC1(s) expression (Fig. 6A). MLC1(1f), MLC1(3f), and MLC1(s) decreased after 48 and 96 h of sepsis (Fig. 6B). Immunoblotting with isoform‐selective antibodies showed that protein levels of MLC1(3f), MLC1(s), and MLC2(f) decreased in the DIA after 24, 48, and 96 h, while levels of MLC1(1f) decreased after 48 and 96 h (Fig. 6C and D).

**Figure 7:**
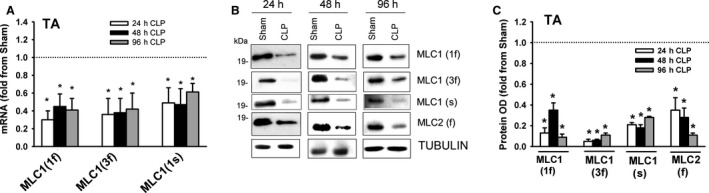
(A) mRNA expressions of myosin light chain isoforms MLC1(1f), MLC1(3f), and MLC1(s) in TA of CLP group. Values are means ± SEM, expressed as fold change from corresponding sham group. *P < 0.05, compared to sham. *N* = 6 per group. (B) Representative immunoblots of MLC1(1f), MLC1(3f), MLC1(s), MLC2(f), and β‐TUBULIN (equal loading indicator) proteins in TA of sham and CLP groups. (C) Protein optical densities of total MLC1(1f), MLC1(3f), MLC1(s), and MLC2(f) in TA of CLP group. Values are means ± SEM, expressed as fold change from corresponding sham group. *P < 0.05, compared to sham. *N* = 6 per group.

In the TA of the sham group, mRNA expression of MLC1(1f) was relatively more abundant than MLC1(3f) or MLC1(s) expression (Fig. 6A). MLC1(1f) expression was higher in the TA than in the DIA, whereas MLC1(s) was lower (Fig. 6A). MLC1(1f), MLC1(3f), and MLC1(s) decreased in the TA after 24, 48, and 96 h of sepsis (Fig. 7A). MLC1(1f), MLC1(3f), MLC1(s), and MLC2(f) protein levels decreased after 24, 48, and 96 h of sepsis (Fig. 7B and C).

### Troponin expression

**Figure 8:**
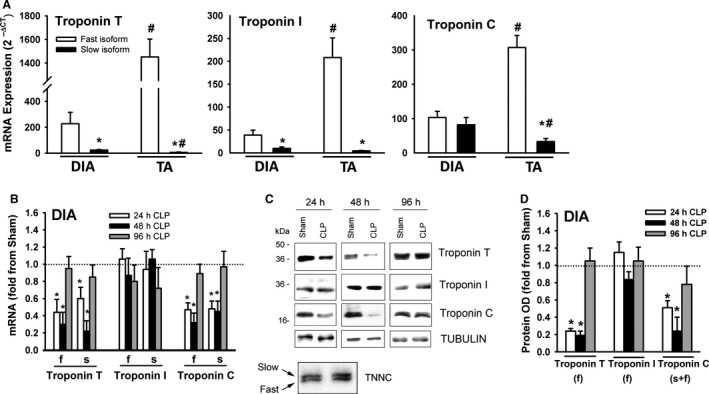
(A) mRNA levels of fast and slow isoforms of troponin T, troponin I, and troponin C in DIA and TA muscles of sham group. Data expressed as absolute values. *P < 0.05, compared to fast isoform; #*P* < 0.05, compared to corresponding isoform in the DIA. (B) mRNA expressions of fast (f) and slow (s) isoforms of troponin T, troponin I, and troponin C in DIA of CLP group. Values are means ± SEM, expressed as fold change from corresponding sham group. *P < 0.05, compared to sham. *N* = 6 per group. (C) Representative immunoblots of troponin T, troponin I, troponin C, and β‐TUBULIN (equal loading indicator) proteins in DIA of sham and CLP groups. (D) Protein optical densities of fast isoforms of troponin T and troponin I and fast and slow isoforms of troponin C in DIA of CLP group. Values are means ± SEM, expressed as fold change from corresponding sham group. *P < 0.05, compared to sham. *N* = 6 per group.

Troponin is a complex of regulatory proteins that consists of three subunits (troponin T, troponin I, and troponin C) that play important roles in the interaction between myosin and α‐Actin. Troponin T (36 kDa) anchors troponin I (21 kDA) and troponin C (18 kDa) to α‐Actin and binds to tropomyosin. Troponin I inhibits actomyosin ATPase; tropomyosin enhances this inhibition. There are two isoforms of troponin C, slow and fast (Lin et al., 2017). Binding of Ca^2+^ to troponin C triggers conformational changes in its structure that alter its interactions with troponin I and troponin T (Potter and Gergely, 1974). In the DIA of the sham group, mRNA expressions of troponin T(f) and troponin I(f) were relatively more abundant than were their corresponding slow isoforms; there was no difference in the expression levels of troponin C(f) and troponin C(s) mRNA (Fig. 8A). Troponin T(f), troponin T(s), troponin C(f), and troponin C(s) decreased in the DIA after 24 and 48 h of sepsis (Fig. 8B). Sepsis had no effect on troponin I(f) and troponin I(s) mRNA levels (Fig. 8B). Immunoblotting with isoform‐selective antibodies detected the fast isoforms of troponin T, troponin I and the fast and slow isoforms of troponin C (Fig. 8C). Troponin T and troponin C protein levels decreased after 24 and 48 h of sepsis; troponin I protein levels were not altered by sepsis (Fig. 8C and D).

**Figure 9:**
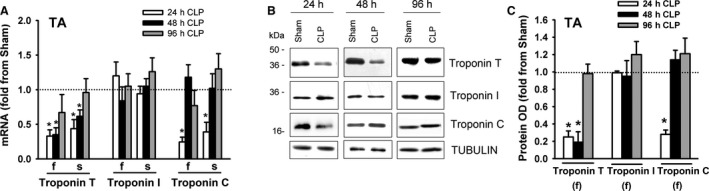
(A) mRNA expressions of fast (f) and slow (s) isoforms of troponin T, troponin I, and troponin C in TA of CLP group. Values are means ± SEM, expressed as fold change from corresponding sham group. *P < 0.05, compared to sham. *N* = 6 per group. (B) Representative immunoblots of troponin T, troponin I, troponin C, and β‐TUBULIN (equal loading indicator) proteins in TA of sham and CLP groups. (C) Protein optical densities of fast isoforms of troponin T and troponin I and fast and slow isoforms of troponin C in TA of CLP group. Values are means ± SEM, expressed as fold change from corresponding sham group. *P < 0.05, compared to sham. *N* = 6 per group.

In the TA of the sham group, mRNA expressions of the fast isoforms of troponin T, troponin I and troponin C were relatively more abundant than were their corresponding slow isoforms (Fig. 8A). mRNA levels of the fast isoforms of troponin T, troponin I and troponin C were higher in the TA than in the DIA (Fig. 8A). mRNA levels of the fast and slow isoforms of troponin T and troponin C decreased in the TA after 24 and 48 h of sepsis (Fig. 9A). Sepsis had no effect on the mRNA levels of the fast or slow isoforms of troponin I (Fig. 9A). Immunoblotting with isoform‐selective antibodies detected the fast isoforms of troponin T, troponin I, and troponin C proteins in the TA (Fig. 9B). Troponin T and troponin C protein levels decreased after 24 and 48 h, but sepsis had no effect on troponin I protein levels (Fig. 9B and C).

### Tropomyosin expression

**Figure 10:**
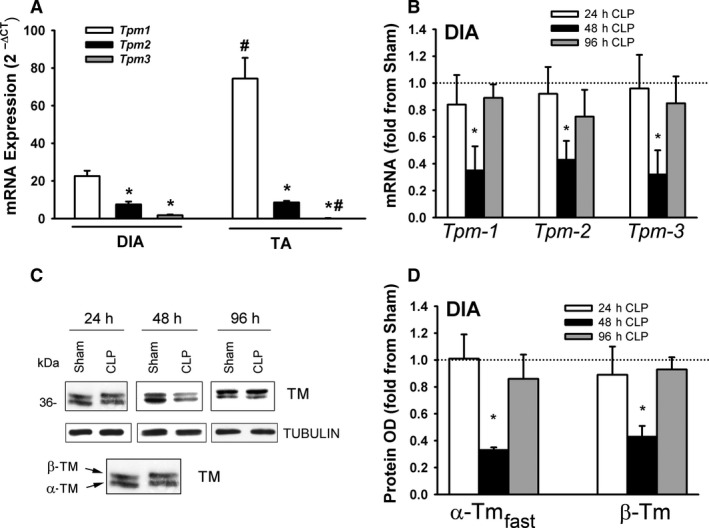
(A) mRNA levels of tropomyosin 1, 2, and 3 isoforms in DIA and TA muscles of sham group. Data expressed as absolute values. *P < 0.05, compared to Tpm1; #*P* < 0.05, compared to corresponding isoform in the DIA. (B) mRNA expressions of tropomyosin isoforms (Tpm‐1, Tpm‐2, and Tpm‐3) in DIA of CLP group. Values are means ± SEM, expressed as fold change from corresponding sham group. *P < 0.05, compared to sham. *N* = 6 per group. (C) Representative immunoblots of tropomyosin (TM) and β‐TUBULIN (equal loading indicator) proteins in DIA of sham and CLP groups. (D) Protein optical densities of tropomyosin isoforms (α‐tropomyosin_fast_ and β‐tropomyosin) in DIA of CLP group. Values are means ± SEM, expressed as fold change from corresponding sham group. *P < 0.05, compared to sham. *N* = 6 per group.

**Figure 11:**
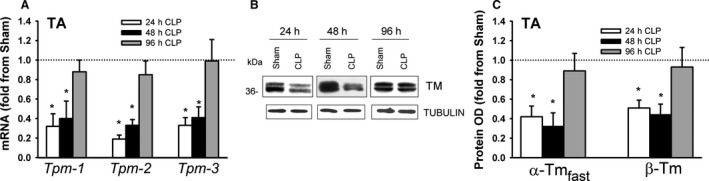
(A) mRNA expressions of tropomyosin isoforms (Tpm‐1, Tpm‐2, and Tpm‐3) in TA of CLP group. Values are means ± SEM, expressed as fold change from corresponding sham group. *P < 0.05, compared to sham. *N* = 6 per group. (B) Representative immunoblots of tropomyosin (TM) and β‐TUBULIN (equal loading indicator) proteins in TA of sham and CLP groups. (C) Protein optical densities of tropomyosin isoforms (α‐tropomyosin_fast_ and β‐tropomyosin) in TA of CLP group. Values are means ± SEM, expressed as fold change from corresponding sham group. *P < 0.05, compared to sham. *N* = 6 per group.

Tropomyosin is an integral component of the protein α‐Actin. Four transcripts have been detected in the skeletal muscles of mammals, including α‐tropomyosin_fast_ (encoded byTpm1), α‐tropomyosin_slow_ (encoded by Tpm3), β‐tropomyosin (encoded by Tpm2), and tropomyosin 4 (encoded by Tpm4) (Gunning et al., 1990). Tpm1 and Tpm3 are expressed in fast and slow twitch muscle fibers respectively. Tpm2 and Tpm4 are expressed in both fast and slow twitch fibers. mRNA expressions of Tpm1, Tpm2, and Tpm3 were detected in the DIA and TA of sham and CLP groups. Tpm1 mRNA levels were relatively more abundant than those of Tpm2 or Tpm3, and were higher in the TA than in the DIA (Fig. 10A). Tpm1, Tpm2, and Tpm3 mRNA levels decreased in the DIA after 48 h of sepsis (Fig. 10B). α‐Tropomyosin_fast_ and β‐tropomyosin proteins were detected in sham and CLP groups by immunoblotting with isoform‐selective antibodies, but α‐tropomyosin_slow_ was not, probably due to its relatively low abundance (Fig. 10C). α‐Tropomyosin_fast_ and β‐tropomyosin protein levels decreased in the DIA after 48 hr of sepsis (Fig. 10C and D). Tpm1, Tpm2, and Tpm3 mRNA levels decreased in the TA after 24 and 48 h of sepsis (Fig. 11A). As was the case in the DIA, immunoblotting detected α‐tropomyosin_fast_ and β‐tropomyosin, but not α‐tropomyosin_slow_ proteins in the TA. Both decreased after 24 and 48 h of sepsis (Fig. 11B and C).

## Discussion

The most important findings of this study are that: (1) the DIA develops transient atrophy, lasting 24 h post‐initiation of sepsis, whereas the TA develops sustained atrophy, persisting for 96 h, (2) atrophy in both muscles is associated with increased expression of ubiquitin E3 ligases and autophagy‐related genes; (3) sepsis elicits time‐dependent and muscle‐ and protein‐specific effects on myofibrillar protein levels, with MLC isoforms decreasing throughout the time course of sepsis in both the DIA and TA, MHC isoforms decreasing early in sepsis in the DIA and throughout the time course in the TA, troponins T and C and tropomyosin decreasing after 24 and 48 h of sepsis in both the DIA and TA, and α‐Actin and troponin I remaining unaffected by sepsis; and (4) sepsis‐induced decreases in myofibrillar protein levels coincide with decreased mRNA expressions of these proteins, suggesting that transcriptional inhibition is involved.

### Sepsis‐induced fiber atrophy

The current study indicates that in the CLP‐induced model of sepsis in mice, DIA fiber atrophy is transient, lasting 24 h after sepsis is initiated, and TA fiber atrophy is sustained, lasting 96 h. These results suggest that the DIA is less sensitive than the TA to the inhibitory effects of CLP‐induced sepsis on fiber size and are in accordance with the work of Prentice et al. (2010), who concluded that the ventilatory muscles are relatively spared from the damaging effects of sepsis in comparison to the limb muscles. Several other studies using the CLP model of sepsis in rats and mice give added weight to this view in that they demonstrate that oxidative stress levels, protein breakdown, and fiber atrophy are significantly lower in the DIA than in limb muscles (Tiao et al., 1997; Morel et al., 2017; Stana et al., 2017; Talarmin et al., 2017). The mechanisms involved in the relative resistance of the DIA to sepsis‐induced fiber atrophy in our model remain unclear. We speculate that DIA fiber composition and its rhythmic pattern of contraction may be behind this relative sparing of sepsis‐induced fiber atrophy. In mice, we found that levels of the fast isoforms of MHC, MLC, troponin T, troponin I, troponin C, and tropomyosin are lower in the DIA than in the TA. Previous reports have indicated that sepsis‐induced protein degradation and metabolic defects are much more severe in muscles rich in glycolytic fibers as compared to those rich in oxidative fibers (Angeras et al., 1991; Tiao et al., 1997).

### Inhibition of muscle protein synthesis

Skeletal muscle protein synthesis is regulated at both the transcriptional and post‐transcriptional levels. Post‐transcriptional regulation is mediated by a balance between the IGF‐1/PI‐3 kinase/protein kinase B (AKT) pathway, which acts as positive regulator of protein synthesis, and the myostatin/SMAD2/3 pathway, which acts as a negative regulator of protein synthesis (Sandri, 2008). AKT promotes protein synthesis primarily through activation of the complex 1 of mTOR (mTORC1). At the same time, AKT also suppresses protein degradation by inhibiting mobilization of FOXO transcription factors (Sandri, 2008). Our group has recently reported that protein phosphorylation of AKT, RPS6K1, and 4E‐BP1 is significantly attenuated in the DIA and TA of mice after 24 and 48 h of CLP‐induced sepsis (Stana et al., 2017). These results indicate that, overall, protein synthesis is indeed inhibited in septic skeletal muscles, most likely through post‐transcriptional mechanisms. Yet, despite it being well‐recognized that general inhibition of protein synthesis occurs in sepsis, little is known about how sepsis affects the transcriptional regulation of myofibrillar proteins.

We found that sepsis triggers gene‐, time‐, and muscle‐specific dependent decreases in mRNA expressions of several myofibrillar genes, suggesting that depressed myofibrillar protein levels may be due, in part, to inhibited transcription. For instance, in the DIA, MHCIIa and MHCIIx mRNA levels decreased after 24 h of sepsis, MHCIIb levels decreased after 24 and 48 h, but MHCI levels remained unchanged. In the TA, mRNA levels of all four MHC isoforms decreased after 24 h of sepsis. The mechanisms responsible for this differential downregulation remain unclear. We speculate that pro‐inflammatory cytokines that are upregulated in septic skeletal muscles, including tumor necrosis factor (TNF‐α) and interferon gamma (IFN‐γ), may be involved, particularly in relation to MHC isoforms. This is because TNF‐α and IFN‐γ inhibition of MHC expression is mediated through activation of the transcription factor NFκB, which selectively inhibits myogenic differentiation factor (MyoD) (Guttridge et al., 2000) and, in doing so, dampens its positive regulatory effect on MHC expression. Moreover, four and a half LIM domains protein 3 (FHL3) may act as a transcriptional co‐activator of MHCIIa and MHCIIb or a co‐repressor of MHCI expression through selective interactions with the transcription factors MyoD and CREB (Zhang et al., 2016). Myocyte enhancer factor 2 (MEF2), serum response factor (SRF), and Kruppel‐like factor 15 (KLF15) are also transcription factors that differentially regulate MHC expression (Harrison et al., 2011; Wang et al., 2014). Alterations in their activities may also trigger the downregulation of myofibrillar gene expression, but this possibility remains to be investigated.

### Differential regulation myofibrillar gene expression in sepsis

Much evidence exists that shows that the calpain, caspase, proteasome, and autophagy proteolytic pathways are significantly induced in the ventilatory and limb muscles of humans and experimental animals with sepsis (Tiao et al., 1994; Hobler et al., 1999; Wei et al., 2005; Klaude et al., 2007; Stana et al., 2017). Despite this evidence, the degree to which these degradation pathways target myofibrillar proteins remains unclear. The proteasome is capable of degrading free myosin and α‐Actin proteins, but it is not capable of degrading intact myofibrils. Myofibrils are initially degraded by calpains and caspases and the myofibrillar proteins that are released are then degraded by the proteasome (Solomon and Goldberg, 1996). In this final step, protein ubiquitination is selectively achieved by specific ubiquitin E3 ligases. For instance, MLC1 and MLC2 are first degraded by the proteasome through MuRF1‐dependent ubiquitination. Upon disassembly of the myofibrils, MuRF1 mediates MHC and troponin degradation, but is not required for α‐Actin degradation (Cohen et al., 2009). α‐Actin is ubiquitinated by Trim32(Cohen et al., 2012). Some evidence suggests that MHC is also ubiquitinated by atrogin‐1(Lokireddy et al., 2012).

The current study is the first to provide comprehensive information on how sepsis affects myofibrillar protein levels over an extended time course of sepsis (24, 48, and 96 h post‐CLP). We found that sepsis differentially decreases individual myofibrillar proteins in a time‐dependent and muscle‐specific fashion. For instance, protein levels of MHC isoforms decreased in the DIA only at 24 h of sepsis, followed by a rapid recovery (Fig. 4), while in the TA MHCs decreased throughout the time course of sepsis (Fig. 5). MLC isoform levels, in contrast, exhibited the same time‐dependent decreases in the DIA as they did in the TA (Figs. 6 and 7). Troponin T, troponin C, and tropomyosin protein levels also decreased significantly in the DIA and TA, but neither α‐Actin nor troponin I in either muscle was affected by sepsis.

As discussed above, it is likely that sepsis‐induced changes in myofibrillar protein levels are mediated both by decreased synthesis and increased degradation. Since myofibrillar protein degradation is ultimately dependent on the proteasome pathway, we surmise that differential expressions of these proteins in the DIA and TA might be due to differences in the degree and time course to which ubiquitin E3 ligases and proteasome activity are induced in these muscles. Indeed, in our recent study, we reported that proteasome activity in the DIA mildly increased after 24 and 48 h of sepsis whereas in the TA it increased more markedly after 48 and 96 h. Levels of atrogin‐1 and MuRF1, two ubiquitin E3 ligases that ubiquitinate MHC, MLC, and troponins, increased to greater levels in the TA than in the DIA (Stana et al., 2017). These results were confirmed in the current study, indicating that thick filament myofibrillar proteins and troponins are likely to be degraded to a more severe extent in the TA than in the DIA.

Our study also showed that α‐Actin mRNA and protein levels in the DIA and TA are unaltered in sepsis. This preferential downregulation and increased degradation of myosin and sparing of α‐Actin is not unique to sepsis; it has been observed in response to aging, immobilization, cancer cachexia, and in patients with myopathies brought on by critical illness (D'Antona et al., 2003; Acharyya et al., 2004; Friedrich et al., 2015). The selective effects of pro‐inflammatory cytokines such as TNF‐α and IFN‐γ are thought to be responsible, not only in relation to their roles in the transcription of myosin heavy and light chain isoforms, but also in relation to the activation of ubiquitination and subsequent degradation by the proteasome (Acharyya et al., 2004).

The current study provides important further evidence of the sparing of α‐Actin in sepsis. It has recently been demonstrated that contractile proteins are degraded in an orderly fashion – thick filament proteins undergo MuRF1‐mediated ubiquitination first, then thin filament proteins are ubiquitinated by Trim32 (Cohen et al., 2012). α‐Actin is one of the thin filament proteins that are ubiquitinated by Trim32. Its degradation is preceded by phosphorylation and ubiquitination of desmin, a muscle architecture protein that maintains the integrity of thin filaments. Upregulation of Trim32 facilitates the breakdown of Z‐bands, Desmin, and, ultimately, α‐Actin by the proteasome during denervation‐induced muscle atrophy (Volodin et al., 2017). We found no significant inductions of Trim32 in the DIA or TA during sepsis, and little is known about its expression or importance in sepsis‐induced skeletal muscle atrophy. Kudryashova et al. demonstrated that Trim32 expression in mouse brain is 100 times that in skeletal muscles and muscle atrophy of Trim32 knockout mice involves both neurogenic and myogenic mechanisms (Kudryashova et al., 2009). While it remains to be explored how these observations can facilitate our understanding of why α‐Actin is relatively spared in sepsis‐induced proteolysis, it is clear that myofibrillar proteins, including Actin, are differentially regulated in septic skeletal muscles and that they represent important new targets for the development of preventative therapies.

### Limitations of the study

Although we report novel information regarding the differential effects of sepsis on the expression of various myofibrillar proteins in skeletal muscles, we acknowledge that our study has two major limitations. First, on the basis of increased LC3B protein lipidation and upregulation of the expression of several autophagy‐related genes and ubiquitin E3 ligases, we report that the autophagy and proteasome proteolytic pathways are activated in the DIA and TA during the course of sepsis (Fig. 3). We did not, however, measure actual protein degradation in skeletal muscles. The exact contributions of the autophagy and proteasome pathways to total protein degradation in the DIA and TA during the course of CLP‐induced sepsis, therefore, remain speculative. Second, we provided no clear mechanisms for the relative resistance of the DIA to sepsis‐induced fiber atrophy and the relatively weak induction of the autophagy and proteasome pathways in that muscle during the course of sepsis. Identification of these mechanisms is beyond the scope of this study. Future studies designed to elucidate the importance of factors such as pro‐inflammatory cytokines and chemokines, fiber type composition, and the pattern of muscle activation in the development of sepsis‐induced fiber atrophy are thus warranted.

In summary, our study indicates that sepsis elicits transient fiber atrophy in the DIA and prolonged atrophy in the TA. In addition, we hypothesize that sepsis‐induced muscle atrophy is mediated by both decreased transcription and increased degradation of several myofibrillar proteins that constitute a significant proportion of total muscle fiber protein, including myosin heavy chain, myosin light chain, troponins T and C, and tropomyosin.

## Conflict of Interest

None declared
